# A Case-control Study of Asthma Death and Life-threatening Attack: Their Possible Relationship with Prescribed Drug Therapy in Japan

**DOI:** 10.2188/jea.12.223

**Published:** 2007-11-30

**Authors:** Shinichi Tanihara, Yosikazu Nakamura, Takehiko Matsui, Sankei Nishima

**Affiliations:** 1 Department of Environmental Medicine, Shimane Medical University.; 2 Department of Public Health, Jichi Medical School.; 3 Department of Pediatrics, Tokyo Metropolitan Ebara Hospital.; 4 National Minami-Fukuoka Chest Hospital.

**Keywords:** asthma, asthma death, life-threatening attack, β_2_-agonist, metered dose inhaler

## Abstract

Sales of inhaled β_2_-agonist bronchodilators may be related to the increase in asthma deaths. The aim of this study is to find whether prescribed drug therapy was associated with the increased risk of death from asthma and life-threatening attacks (LTA). The “case” group comprised those under 35 years of age who expired or experienced LTA from January 1994 through December 1996. For each case, an age and sex matched control was selected from asthma patients. Hospital records were reviewed to obtain information on the prescribed drug therapy and clinical asthma severity for the cases and controls. Bivariate analysis with conditional logistic regression models for matched data sets were used to estimate the severity-adjusted odds ratios for each asthma medication. Twenty-four fatal cases and 54 LTA cases were observed. The crude odds ratio of clinical severity (OR=9.33, 95%CI:2.84-30.7) was larger than unity and with statistical significance. After adjusting for clinical severity, the odds ratios computed for all β_2_-agonists delivered by metered dose inhaler (MDI) increased (OR=2.08, 95%CI:0.78-5.50) from that of crude analysis. Among those subjects under 20 years of age, the clinical severity-adjusted odds ratio for the use of all β_2_-agonists by MDI (OR=3.67, 95%CI:0.77-17.5) was higher than that of all subjects. The prescription of β_2_-agonists by MDI increased the risk of asthma death after taking clinical severity into account. Although not statistically significant, our results suggested that β_2_-agonists administered by a MDI might have increased the risk of asthma death and LTA in Japan because the magnitude of the effect was similar to that reported in other countries.

## INTRODUCTION

In the 1960s, asthma mortality was alluded to during an epidemic on the basis of an ecologic study in England and Wales^1^^,^^2^^)^ and in Japan^3^^)^, suggesting that sales of inhaled β_2_-agonist bronchodilators may have been related to excessive asthma deaths during the epidemic. In the 1980s, a relation between inhaled fenoterol and increased risk of death from asthma was reported from New Zealand^4^^-^^7^^)^. However, these findings might be due to a tendency to prescribe the drug more frequently for patients with severe asthma^8^^)^.

Many investigations were conducted to elucidate uncertainty about the confounding between severity and fenoterol use^4^^-^^11^^)^. Opinion is still divided: one is that fenoterol was not associated with severe life-threatening attacks (LTA) after controlling severity^8^^,^^10^^)^; and the other is that the association between fenoterol and asthma death was significant, but after adjustment for markers of asthma severity was made, there was no difference in the magnitude of the association between fenoterol and asthma death^4^^-^^7^^,^^9^^,^^11^^)^. In this paper, we report the results of a case-control study on prescribed drug therapy, severity, and the risk of death from asthma and LTA in Japan.

## METHODS

### Case and Control Selection

In January 1998, a questionnaire on the clinical courses of asthma patients (LTA or death from asthma) was mailed to the departments of internal medicine, respiratory medicine, allergy and collagen diseases, and pediatrics of hospitals with 100 or more beds throughout Japan. The criteria for eligible cases were: 1) asthma patients aged under 35 with LTA or death from asthma during a three-year period from January 1994 through December 1996 and 2) under asthma therapy at the department of the hospital during a one-year period before LTA or death. LTA is defined as requiring airway intubation or assisted ventilation and resulting in a loss of consciousness due to asthma. Of the 8,454 departments, 2,711 responded and 466 reported that they had treated qualifying cases.

In January 1999, physicians working in the 466 department that have treated qualifying cases were asked whether they had also treated eligible controls. A control was defined as one that required asthma treatment from the department of the hospital during the one-year period but who had never experienced LTA until the time the case subject experienced it or expired. If the department had both eligible cases and controls, one eligible control was selected for each eligible case. The control was matched for age (within 3 years) and sex. If there were two or more eligible controls in regard to age, the nearest age control was selected. If a suitable control could not be found, the selection period was extended to include 1994 through 1996. If a suitable control was still unavailable, a control of the opposite sex was selected.

### Information on Prescribed Drug Treatment and Clinical Asthma Severity

Regardless of the case-control status, hospital records were reviewed to obtain information on the prescribed drug therapy and clinical asthma severity by the physicians who had reported the eligible cases and controls. The principal risk factor examined was the use of inhaled β_2_-agonists delivered by a metered-dose inhaler (MDI). Exposure to a certain drug is defined as whether a drug was prescribed for the patient during the one-month period prior to the date of his or her demise or the development of LTA.

To assess clinical asthma severity, criteria that consisted of clinical features and that were based on the International Consensus Report on the Diagnosis and Treatment of Asthma were chosen^12^^)^. In this study, clinical asthma severity was dichotomized into ‘severe’ and ‘non-severe’ groups. The former included those under 20 years of age and with a history of major asthma attacks during the one-year period prior to the date of death or LTA. The group also includes those 20 years or older who experienced exacerbation of asthma affecting physical activity or sleep, and whose activities of daily living were restricted prior to the same period. The subjects with no experience of a major asthma attack during the period were placed in the ‘non-severe’ group. Those who were asymptomatic or between exacerbations during the period were also placed in this group.

### Data Analysis

Conditional logistic regression models for matched data sets were used to estimate the odds ratios for the effects of asthma medications. The categories of asthma medications are : 1) β_2_-agonists by MDI, 2) β_2_-agonists by nebulizers, 3) oral β_2_-agonists, 4) oral theophyllines, 5) sodium cromoglycate, 6) inhaled corticosteroids, and 7) oral corticosteroids. The risk of particular β_2_-agonists (fenoterol, salbutamol, and procaterol by MDI and salbutamol and procaterol by nebulizer) was estimated independently. Data were analyzed with the aid of the SAS statistical package (Version 6.12). Initially, univariate analyses were carried out to estimate the crude effect of each variable. Next, bivariate analyses were performed to estimate the severity-adjusted odds ratio for each asthma medication. The possibility of effect modification by age was assessed by calculating the odds ratios for two subgroups categorized by age. One group consisted of patients under 20 years of age and the other, those whose ages were 20 years or older.

## RESULTS

Twenty-four fatal cases, 54 with LTA, and 78 controls were observed. Table 1 shows the age distribution of these subjects. No age discrepancy was noted in any of the case and control pairs (both the case and control in each pair being under or other over 20 years). Among those under 20 years, there were 14 fatal cases and 32 with LTA. In the group aged 20 years or older, there were 10 fatalities and 22 developing LTA.

**Table 1.** Age distribution of study subjects.

age (year)	Cases	Controls
0-4	12	12
5-9	9	9
10-14	15	18
15-19	10	8
20-24	9	10
25-29	16	14
30-35	7	7
Total	78	78

The frequency of administering asthma medications and clinical severity are shown in an unmatched format for the cases and the controls, together with the calculated crude odds ratios (Table 2). β_2_-agonists by MDI were prescribed for 51% of the cases and 41% of the controls. Fenoterol by MDI was prescribed for 17% of the cases and 15% of the controls. The crude odds ratios indicated that β_2_-agonists by MDI [fenoterol (OR=1.14, 95%CI:0.41-3.15), salbutamol (OR=1.67, 95%CI:0.40-6.97) and procaterol (OR=1.67, 95%CI:0.61-4.59)] did not augment the risk of an asthma death or LTA with statistical significance.

**Table 2.** Prescribed medication, clinical severity, and the relative risk of asthma death or life threatning attack (total subjects).

Exposure	Cases		Controls		Crude	95%CI	p value
	Yes	No	Yes	No	Odds ratio		
β_2_ agonists by MDI	40	38	32	46	1.89	0.84 - 4.24	0.12
(Fenoterol)	13	65	12	66	1.14	0.41 - 3.15	0.80
(Salbutamol)	8	70	6	72	1.67	0.40 - 6.97	0.48
(Procaterol)	17	61	13	65	1.67	0.61 - 4.59	0.32
β_2_ agonists by nebuliser	30	48	23	55	1.61	0.81 - 3.23	0.17
(Salbutamol)	9	69	12	66	0.70	0.27 - 1.84	0.47
(Procaterol)	16	62	11	67	2.25	0.69 - 7.31	0.18
Oral β_2_ agonists	43	35	48	30	0.67	0.30 - 1.48	0.32
Oral theophyllines	54	24	63	15	0.50	0.23 - 1.11	0.09
Sodium cromoglycate	31	47	31	47	1.00	0.52 - 1.92	1.00
Inhaled corticosteroids	19	59	21	57	0.88	0.44 - 1.77	0.72
Oral corticosteroids	12	66	7	71	2.00	0.68 - 5.85	0.21
Clinical severity	38	40	13	65	9.33	2.84 - 30.7	<0.01

(Exposure to asthma medication assessed one month prior to the event.)

95%CI: 95% confidence interval; MDI: metered dose inhaler.

β_2_-agonists by a nebulizer were prescribed for 38% of the cases and 29% of the controls. Procaterol by a nebulizer (OR=2.25, 95%CI:0.67-7.31) increased the risk of an asthma death or LTA more than twice; but the increase was not statistically significant. More than half of the subjects had oral β_2_-agonists (55% of the cases and 62% of the controls) and oral theophyllines (69% of the cases and 81% of the controls) prescribed. Those drugs-oral β_2_-agonists (OR=0.67, 95%CI:0.30-1.48) and oral theophyllines (OR=0.50, 95%CI:0.23-1.11)–reduced the risk but the data were not statistically significant. Sodium cromoglycate was prescribed for 40% of both cases and controls but it did not change the risk (OR=1.00, 95%CI:0.52-1.92). The proportion of subjects who had inhaled the prescribed corticosteroids was higher than for those on oral corticosteroids among both cases (24% by inhaling, 15% by oral) and controls (27% by inhaling, 9% by oral). Inhaled corticosteroids (OR=0.88, 95%CI:0.44-1.77) showed a null value for the risk of an asthma death or LTA. Oral corticosteroids (OR=2.00, 95%CI:0.68-5.85) increased the risk but the result was not statistically significant. The proportion of cases with clinically severe asthma was higher (49%) than the controls (17%). The odds ratio for clinical severity (OR=9.33, 95%CI:2.84-30.7) was higher than unity, with statistical significance.

The severity-adjusted odds ratios for each asthma medication, using bivariate analysis, are shown in Table 3. The association between the prescription of an asthma drug and the risk of an asthma death or LTA changed when univariate analysis was used. The odds ratios of asthma drugs were less than unity, except the inhaled β_2_-agonists by MDI. The odds ratio of oral corticosteroids decreased from 2.00 to 0.65 when the clinical severity was adjusted. Oral β_2_-agonists (OR=0.36, 95%CI:0.13-0.98) and oral theophyllines (OR=0.40, 95%CI:0.16-0.98) reduced the risk with statistical significance. Salbutamol administered by a nebulizer (OR=0.26, 95%CI:0.06-1.09) reduced the risk but without significance. Among β_2_-agonists delivered by MDI, the severity-adjusted odds ratios of fenoterol (OR=2.18, 95%CI:0.56-8.55) and salbutamol (OR=2.74, 95%CI:0.36-20.8) were higher than the crude odds ratios.

**Table 3.** Severity adjusted odds ratios from bivariate analysis for prescribed medication (total subjects).

Exposure	Adjusted Odds ratio	95%CI	p value
β_2_ agonists by MDI	2.08	0.78 - 5.50	0.14
(Fenoterol)	2.18	0.56 - 8.55	0.26
(Salbutamol)	2.74	0.36 - 20.8	0.33
(Procaterol)	0.85	0.25 - 2.87	0.07
β_2_ agonists by nebuliser	0.72	0.29 - 1.75	0.46
(Salbutamol)	0.26	0.06 - 1.09	0.07
(Procaterol)	1.05	0.25 - 4.43	0.95
Oral β_2_ agonists	0.36	0.13 - 0.98	0.05
Oral theophyllines	0.40	0.16 - 0.98	0.05
Sodium cromoglycate	0.79	0.38 - 1.67	0.54
Inhaled corticosteroids	0.65	0.27 - 1.57	0.34
Oral corticosteroids	0.84	0.20 - 3.59	0.81

(Exposure to asthma medication assessed one month prior to the event.)

95%CI: 95% confidence interval; MDI: metered dose inhaler.

The severity-adjusted odds ratios of each asthma medication given to the subjects under 20 years of age were estimated by bivariate analysis. The frequency of asthma medications and severity-adjusted odds ratios are shown in Table 4. The crude odds ratio of clinical severity (19 cases and 7 controls, OR=13.0, 95%CI: 1.70-99.4) indicated a statistically significant increased risk for all of the subjects. The severity-adjusted odds ratios of all β_2_-agonists (OR=3.67, 95%CI: 0.77-17.5), fenoterol administered (OR=3.10, 95%CI:0.33-29.4), and salbutamol(OR=4.28, 95%CI:0.27-66.8), all delivered by MDI, were greater than those for all of the subjects, although the differences were not statistically significant. Salbutamol administered by a nebulizer (OR=0.12, 95%CI:0.01-1.01) decreased the risk but the difference was not statistically significant.

**Table 4.** Prescribed medication and severity adjusted odds ratios from bivariate analysis (subjects under 20 years of age).

Exposure	Cases		Controls		Adjusted Odds ratio	95%CI	p value
	Yes	No	Yes	No			
β_2_ agonists by MDI	18	28	10	36	3.67	0.77 - 17.5	0.10
(Fenoterol)	4	42	1	45	3.10	0.33 - 29.4	0.32
(Salbutamol)	3	43	3	43	4.28	0.27 - 66.8	0.30
(Procaterol)	9	37	6	40	0.80	0.11 - 5.99	0.82
β_2_ agonists by nebuliser	22	24	20	26	0.41	0.13 - 1.32	0.13
(Salbutamol)	6	40	10	36	0.12	0.01 - 1.01	0.05
(Procaterol)	14	32	11	35	0.87	0.18 - 4.19	0.86
Oral β_2_ agonists	29	17	31	15	0.50	0.14 - 1.73	0.27
Oral theophyllines	34	12	39	7	0.40	0.13 - 1.19	0.10
Sodium cromoglycate	21	25	20	46	1.13	0.43 - 3.02	0.80
Inhaled corticosteroids	12	34	6	40	2.00	0.46 - 8.32	0.37
Oral corticosteroids	2	44	3	43	0.23	0.02 - 3.43	0.29

(Exposure to asthma medication assessed one month prior to the event.)

95%CI: 95% confidence interval; MDI: metered dose inhaler.

Table 5 shows the clinical severity-adjusted odds ratios obtained by bivariate analysis and the frequency of asthma medications given to subjects aged 20 years and older. In the crude analysis, the clinical severity (19 patients and 6 controls, OR=7.50, 95%CI: 1.72-32.8) had an increased statistically significant risk that was analogous to all of the subjects and those below the age of 20. Although without statistical significance, oral β_2_-agonists (OR=0.22, 95%CI:0.04-1.19), oral theophyllines (OR=0.38, 95%CI:0.08-1.90), sodium cromoglycate (OR=0.49, 95%CI:0.15-1.65), and inhaled corticosteroids (OR=0.49, 95%CI:0.15-1.65) appeared to have decreased the risk. The odds ratios for all β_2_-agonists, all administered by MDI (OR=1.19, 95%CI:0.31-4.54), fenoterol (OR=1.56, 95%CI:0.25-9.73), and salbutamol (OR=1.88, 95%CI:0.07-50.1) were greater than unity, but lower than those of subjects under 20 years. Salbutamol given by a nebulizer (OR=1.26, 95%CI:0.08-19.7) did not decrease the risk, as observed in the subjects under 20 years.

**Table 5.** Prescribed medication and severity adjusted odds ratios from bivariate analysis (subjects 20 years of age or older).

Exposure	Cases		Controls		Adjusted Odds ratio	95%CI	p value
	Yes	No	Yes	No			
β_2_ agonists by MDI	22	10	22	10	1.19	0.31 - 4.54	0.80
(Fenoterol)	9	23	11	21	1.56	0.25 - 9.73	0.64
(Salbutamol)	5	27	3	29	1.88	0.07 - 50.1	0.70
(Procaterol)	8	24	7	25	0.84	0.18 - 2.87	3.93
β_2_ agonists by nebuliser	8	24	3	29	2.50	0.42 - 14.9	0.32
(Salbutamol)	3	29	2	30	1.26	0.08 - 19.7	0.87
(Procaterol)	2	30	0	32	-	- - -	-
Oral β_2_ agonists	14	18	17	15	0.22	0.04 - 1.19	0.08
Oral theophyllines	20	12	24	8	0.38	0.08 - 1.90	0.24
Sodium cromoglycate	10	22	11	21	0.49	0.15 - 1.65	0.25
Inhaled corticosteroids	7	25	15	17	0.28	0.07 - 1.04	0.06
Oral corticosteroids	10	22	4	28	1.89	0.28 - 12.6	0.51

(Exposure to asthma medication assessed one month prior to the event.)

95%CI: 95% confidence interval; MDI; metered dose inhaler.

## DISCUSSION

The aim of this study was to determine whether a prescribed drug therapy was associated with the risk of death from asthma and LTA. The starting hypothesis was that β_2_-agonists delivered by MDI are related to the risk of asthma deaths and LTA after controlling for the clinical severity of asthma. The results suggest that self-administration of β_2_-agonists via MDI inhalation increases the risk of asthma death after accounting for the clinical severity, especially in subjects under 20 years of age.

In other studies, there were three types of parameters for clinical severity of asthma were given: hospital admission in the 12 months prior to asthmatic death of LTA^4^^-^^7^^,^^10^^,^^11^^,^^13^^,^^14^^)^, the use of three or more categories of drugs^4^^-^^7^^,^^10^^,^^11^^,^^13^^,^^14^^)^, and the prescription of oral corticosteroids^4^^-^^7^^,^^9^^,^^10^^,^^11^^,^^13^^,^^14^^)^. In this study, the clinical severity was defined in terms of asthma symptoms and the frequency of exacerbation. Only clinical severity was adjusted in bivariate analysis with conditional logistic regression models for matched data sets. Because the management of asthma is considered to control its symptoms, adjusting the clinical severity of asthma and the pattern of asthma drug administration may simultaneously cause problems in multicolinearity. In discussing the relationship between β_2_-agonists administered by MDI and asthma death, it is necessary to consider severity^15^^)^. In this study, the crude odds ratio for oral corticosteroids decreased when adjustments were made for clinical severity. This indicates that the unadjusted findings were probably largely affected by the confounding effect of clinical severity.

The severity-adjusted odds ratios of β_2_-agonists given by MDI were not statistically significant, although they were larger than unity. It was reported that the entire class of selective β_2_-agonists was associated with an elevated asthma mortality^16^^)^. There were differences among β_2_-agonists administered by MDI vis-à-vis the risk of death from asthma and LTA. After adjusting for the severity, an increased risk with fenoterol^4^^-^^7^^)^ and albuterol^9^^,^^16^^)^, no relationship in salbutamol^4^^,^^6^^)^, salmeterol^13^^)^ and albuterol^17^^)^, and a decreased risk with salbutamol^5^^)^ were reported. In this study, salbutamol when administered by MDI, increased the risk, but when the drug was delivered by a nebulizer, the risk generally decreased. It was reported that salbutamol given by MDI decreased the relative risk of having a near-fatal attack of asthma; but administration of the same drug (salbutamol) via a nebulizer increased the risk^13^^)^. The overall increase in risk for patients on fenoterol administered by MDI (OR=2.18, 95%CI:0.56-8.55) was similar to the results obtained from an investigation conducted in New Zealand^4^^-^^7^^)^. It is necessary to find out if each β_2_-agonist administered by MDI increases the risk of asthma death and LTA.

After adjusting for clinical severity in this study, oral β_2_-agonists and theophyllines decreased the risk. The findings on oral β_2_-agonists have been inconsistent, according to several studies^4^^-^^7^^,^^10^^)^. Most do not generally show an increased risk^4^^,^^5^^,^^7^^,^^10^^)^. However, an increased risk was observed in a cohort analysis^9^^)^, and among subjects who were taking oral corticosteroids at admission^6^^)^. Similar findings were reported for oral theophyllines^4^^-^^6^^,^^14^^)^. Some have shown an increased risk^14^^)^, some have not^4^^-^^6^^)^. Further investigation is required with respect to the way these drugs are used.

The findings for oral corticosteroids, inhaled corticosteroids and β_2_-agonists inhaled by nebulizer were inconsistent among age groups. For β_2_-agonists given by MDI, it was reported that higher risks were observed in subjects under 20 than in their older counterparts^4^^,^^5^^)^. These results are consistent with those obtained in this study. The findings may be due to differences in treatment compliance^18^^)^. It has been reported that compliance to asthma treatment varies with age^19^^)^.

The findings of this study are consistent with the case-control studies conducted in New Zealand^4^^-^^7^^)^ although the results are not statistically significant. The results of a test for statistical significance rely on both the sample size and magnitude of the effect; therefore a statistically significant finding does not always provide substantial evidence in medical or public health practice. A lack of statistical significance does not provide evidence for the absence of an effect^20^^)^. Our results may support the hypothesis that the self-administration of β_2_-agonists given by MDI increases the risk for asthma death even when clinical severity is taken into account.
